# Comparing Effects of Conventionally and Ultrasonically Extracted Flaxseed Mucilages as Sustainable Egg Replacers on the Technological Properties and Nutritional Quality of Dried Wheat Noodles

**DOI:** 10.1002/fsn3.71727

**Published:** 2026-04-02

**Authors:** Elif Yaver

**Affiliations:** ^1^ Department of Food Processing, Vocational School of Technical Sciences Konya Technical University Konya Türkiye

**Keywords:** egg replacer, flaxseed mucilage, noodles, sustainability, ultrasound‐assisted extraction

## Abstract

Eggs are one of the basic ingredients of noodles, providing unique technological and sensory features. Also, eggs are important sources of proteins, vitamins and minerals. Despite excellent technological and nutritional features of eggs, they contain high amounts of saturated and *trans* fatty acids, which are thought to be responsible for some health problems, such as cardiovascular diseases and hypercholesterolemia. In this study, eggs in the formulation of dried wheat noodles were replaced by two types of flaxseed mucilage extracted from meal using conventional method and ultrasound‐assisted extraction. The results illustrated that color L* and a* values of noodle samples increased, but b* values decreased when fully replacing eggs with flaxseed mucilage. The use of sonicated mucilage resulted in higher ΔE (4.20), water uptake (195.5%), volume increase (238.1%) and firmness (5.09 N) values in noodles compared to conventionally extracted ones (3.88%, 193.1%, 217.5%, and 4.85 N, respectively). On the other hand, noodles made with 100% flaxseed mucilage had lower cooking loss (4.42%) and higher firmness (5.82 N) than 100% egg noodles (5.32% and 4.30 N, respectively). Mucilage type had no notable impact on the moisture, ash, protein and carbohydrate contents. Replacing 100% of eggs with flaxseed mucilage decreased the fat content (~84.5%) and energy value and increased carbohydrate content of noodles. Although the protein content of noodles decreased by replacing eggs with mucilage, all noodle samples still had a protein content above 13.5%. Replacing 100% of eggs with mucilage reduced the total phenolic content of samples from 0.43 to 0.39 g GAE/kg. Besides that, both mucilage types demonstrated similar antioxidant properties in noodles. Compared to conventional mucilage (7.39), sonicated mucilage revealed greater overall acceptability scores (7.68). However, replacing 100% of eggs with mucilage slightly reduced the overall acceptability of noodles. The findings suggest that flaxseed mucilage, especially extracted by ultrasonication, is a promising replacer for eggs in the production of acceptable energy‐reduced wheat noodles.

## Graphical abstract

In this study, eggs in the formulation of dried wheat noodles were replaced by two types of flaxseed mucilage extracted from meal using conventional method and ultrasound‐assisted extraction. The study suggests that flaxseed mucilage, especially extracted by ultrasonication, is a promising replacer for eggs in the production of acceptable energy‐reduced wheat noodles.

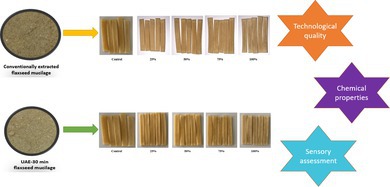

## 1 Introduction

Nowadays, there is an increasing desire among consumers to substitute traditional animal foods with plant‐based alternatives due to ethical, ecological, well‐being, and sustainability considerations (Rokib et al. 2021; Akter et al. 2024; Bhuiyan and Ngadi 2024; McClements and Grossmann 2026). Eggs are one of the most popular animal foods worldwide because of their great amounts of protein with a well‐balanced amino acid composition, vitamins (A, B_1_, B_2_, B_5_, B_9_, and D), and minerals (P, Zn, Fe, and Ca) (Oladimeji and Gebhardt 2023). Moreover, egg proteins may reveal unique emulsifying, gelling, thickening, and foaming characteristics (Zhou et al. 2024). Despite many functional and nutritional advantages of eggs, their high cholesterol and saturated fat content, rising price, and the risks of allergenicity and *Salmonella* further increase the need for plant‐based alternatives (McClements and Grossmann 2026).

Plant‐based proteins, emulsifiers and hydrocolloids are used as egg replacers in several food formulations such as cakes (Kim and Shin 2022), mayonnaise (Nikolova et al. 2023) and salad dressings (de Angelis et al. 2022). As sustainable and native gums, seed mucilages represent a promising source of soluble dietary fibers with excellent functional properties as well as potential health benefits (Singh et al. 2025). Apart from being a source of dietary fiber, mucilages can also be used as a stabilizer, thickener, gelling and emulsifying agent in the food sector (Uyor et al. 2025). Nouri (2025) stated that the incorporation of seed mucilages as a gluten replacer into gluten‐free cake formulation enhanced the textural and rheological quality of the end product. Neeharika and Vijayalaxmi (2024) used basil seed mucilage as a fat replacer to prepare mayonnaise, and they found that the use of basil seed mucilage enhanced antioxidant activity and reduced fat content of mayonnaise samples. However, there are limited studies in the literature on the use of mucilages as an egg replacer in food formulations. Moreira et al. (2023) reported that replacing eggs with chia seed mucilage did not considerably change the technological properties of brownies and also increased their fiber content. In another study, chia mucilage gel was used as an egg replacer in cake formulation. The authors observed that replacing egg yolk with chia mucilage gel in cake formulation showed high viscosity and acceptable sensory properties (Gallo et al. 2020).

Flaxseed ( *Linum usitatissimum* L.) mucilage does not contain cholesterol and any known allergens, which is also a safe, native, sustainable and healthy alternative to eggs (Safdar et al. 2020). Conventionally, flaxseed mucilage was obtained by whole seed/meal or defatted cake by the hot‐water extraction method (Yu et al. 2022). The extraction process has a considerable impact on the yield, functional and physicochemical properties of mucilage (Uyor et al. 2025). The ultrasound‐assisted extraction method offers a faster and more efficient alternative to the conventional method for extracting mucilage (Yeasmen, Bhuiyan, Gariepy, et al. 2025; Yeasmen, Bhuiyan, Ramaswamy, et al. 2025). To date, the effect of ultrasound‐assisted extraction on the yield, functional, chemical and structural properties of flaxseed mucilages has been investigated by some researchers (Safdar et al. 2020; Emadzadeh et al. 2022). Nonetheless, a recent study on gluten‐free noodle quality underscored the impacts of ultrasound‐assisted extracted flaxseed mucilage (Yaver 2026); the impact of sonicated flaxseed mucilage on the quality of traditional cereal‐based foods remains uncovered. Therefore, the aim of the present study is to address that knowledge gap.

The main goals of the present study are (1) to replace eggs in wheat noodles with flaxseed mucilages extracted by conventional and ultrasound‐assisted methods at 0%, 25%, 50%, 75%, and 100% levels, (2) to investigate the impacts of both types of flaxseed mucilages as egg replacers on the physical, technological, chemical, nutritional, and sensory features of wheat noodles, and (3) to compare the mentioned features between noodles prepared with different mucilage types at various levels.

## 2 Materials and Methods

### 2.1 Materials

Commercial wheat ( *Triticum aestivum* L.) flour (contains 13.36% protein, 0.62% ash, and 11.37% moisture; Hekimoğlu, Konya, Türkiye), chicken eggs (Bili Bili, Ankara, Türkiye), and salt were procured from a local store in Konya, Türkiye. Cold‐pressed brown flaxseed ( *Linum usitatissimum* L.) meal was kindly provided by Soivera, Tokat (Türkiye). All chemicals used in the study were of analytical grade.

### 2.2 Extraction of Mucilage

#### 2.2.1 Extraction by Conventional Method

Flaxseed meal was suspended in pure water in a ratio of 1:20 (w/v) and subjected to shaking for 180 min at 50°C. The suspension was filtered and centrifuged at 2071×*g* for 15 min (Nüve NF800, Ankara, Türkiye). One volume of ethanol was added to the supernatant and allowed to stand at 4°C for 60 min. Following that, the precipitate was obtained via centrifugation at 2071×*g* for 10 min and then subjected to drying in a hot‐air oven (Nüve KD200, Ankara, Türkiye) at 45°C until 10% moisture content. The dried sample was pulverized (Fakir Aromatic, Vaihingen, Germany) into powder (≤ 250 μm) (Yu et al. 2022).

#### 2.2.2 Extraction by Ultrasound‐Assisted Method

Flaxseed meal was sonicated (Bandelin Sonorex RK102H, Berlin, Germany) in pure water at a meal‐to‐water ratio of 1:20 (w/v) at 50°C for 30 min. After centrifugation (2071×*g* for 15 min) of the suspension, the supernatant was subjected to the same processes as the conventional method (Yaver et al. 2026).

### 2.3 Wheat Noodle Production

Control wheat noodle formulation consisted of 200 g wheat flour, 60 g whole chicken eggs, 1 g salt and water. The ingredients were subjected to mixing to prepare a homogenous dough in a food processor (Arzum Crust Mix, İstanbul, Türkiye) at speed 2 for 6 min. The dough was kept at room temperature (25°C ± 2°C) for 20 min. Then, it was sheeted (Cooker 2822, İstanbul, Türkiye) and sliced into noodles with a length of 4 cm and a thickness of 2 mm. The samples were dried in the air oven (55°C—20 h; Nüve KD200) (Yaver 2023).

To prepare noodles containing flaxseed mucilages, whole eggs were replaced with the solutions [8 g mucilage in 100 mL pure water (Fernandes and Mellado 2018)] of conventionally extracted flaxseed mucilage (CFM) and ultrasonically extracted flaxseed mucilage (USFM) separately at 25%, 50%, 75%, and 100% levels.

With mucilage type (CFM and USFM) and mucilage level (0%, 25%, 50%, 75%, and 100%) as the two independent variables, the experiments employed a (2 × 5) × 2 factorial design, and noodle preparation was performed in duplicate.

### 2.4 Color Properties

A chroma meter (Konica Minolta CR‐400, Osaka, Japan) was used to measure CIE L* (lightness), a* (redness‐greenness), and b* (yellowness‐blueness) of dry noodle samples. The ΔE value representing the total color difference between the control and mucilage‐containing noodles was calculated as follows (Francis and Clydesdale 1975):

$$∆E=\sqrt{{\left(L_{\text{Sample}}^{*}-L_{\text{Control}}^{*}\right)}^{2}+{\left(a_{\text{Sample}}^{*}-a_{\text{Control}}^{*}\right)}^{2}+{\left(b_{\text{Sample}}^{*}-b_{\text{Control}}^{*}\right)}^{2}}$$

### 2.5 Cooking Properties and Textural Analysis

Measurement of the optimal cooking time, signifying the achievement of full starch gelatinization, was carried out by applying pressure to samples positioned between glass tiles at different stages according to the AACC method 66–50 (data not illustrated) (AACC 2010). For the determination of cooking properties, noodles (20 g) were cooked to optimum in 250 mL of boiling water. Before the assessment of the weight and volume of cooked noodles, they were allowed to drain for 120 s. The water uptake and volume increase values were determined according to the method described by Yaver and Bilgiçli (2021). Cooking loss of noodles was determined using the AACC method 66–50 (AACC 2010). To measure the weight of total solids loss during noodle cooking, the cooking loss, the cooking water was evaporated to dryness at 135°C. After weighing the residue, the cooking loss was expressed as a percentage of uncooked noodle weight.

Firmness of the cooked samples was determined according to the AACC 66–50 method with a texture analyzer (Stable Micro Systems TA‐XT.Plus, Surrey, UK) equipped with an A/LKB‐F probe (AACC 2010). Three strips of noodles were positioned on a plexiglass plate side by side and cut at a 90° angle with the probe. Test parameters were: test speed, 1 mm/s; post‐test speed, 10 mm/s; distance, 4.5 mm; and load cell, 30 kg.

### 2.6 Chemical Quality

The chemical composition of wheat noodles was measured according to the AACC methods (AACC 2010). The AACC methods of 44–19, 08–01, 30–25 and 46–30 were used to determine moisture, total ash, crude fat and crude protein (Nx6.25) contents of wheat noodles, respectively. Total carbohydrate was determined by the difference method [100 − (moisture + ash + fat + protein)]. The formula [(protein × 4 kcal/g) + (fat × 9 kcal/g) + (carbohydrate × 4 kcal/g)] was used to calculate the energy value (kcal/100 g) of samples.

### 2.7 Evaluation of Phenolic Content and Antioxidant Properties

Uncooked dry noodle powders (2 g) and 20 mL of acidified methanol (HCl:methanol:pure water, 1:80:10, v/v/v; Sigma‐Aldrich, St. Louis, MO, USA) were added to tubes and extracted in a shaking water bath (30°C–120 min; Nüve ST30, Ankara, Türkiye). The supernatants were collected following the centrifugation process (Nüve NF800) at 1372×*g* for 10 min (Yaver and Bilgiçli 2021).

The Folin–Ciocalteu method was applied to extracts to determine total phenolic content (Anton et al. 2008). The extract (0.2 mL) and Folin–Ciocalteu reagent (1.5 mL, tenfold‐diluted; Sigma‐Aldrich) were added to the test tube. After 5 min, 1.5 mL of aqueous Na_2_CO_3_ (6%, w/v; Sigma‐Aldrich) was added. The absorbance was measured at 725 nm (Jenway 6100, Essex, UK) after keeping the reaction for 90 min in darkness. Total phenolic content was calculated using a standard curve of gallic acid (Sigma‐Aldrich) and the data were illustrated as gram gallic acid equivalents (GAE) per kg of noodles (on a dry basis).

The 2,2‐diphenyl‐1‐picrylhydrazyl (DPPH) assay was applied to determine the antioxidant activity of noodles according to the method of Anton et al. (2008). The extract (0.2 mL) was reacted with 3.8 mL of 6.34 × 10^−5^ mol/L DPPH solution (Sigma‐Aldrich) and kept for 30 min in darkness. The absorbance was read at 517 nm. The antioxidant activity was expressed as μmol Trolox equivalents (TE) per g of noodles (on a dry basis) using a standard curve of Trolox (Sigma‐Aldrich).

### 2.8 Sensory Quality

Sensory characteristics, including taste, odor, color, appearance, mouthfeel, and overall acceptability, of cooked wheat noodles were assessed by 25 semi‐trained panelists (13 female and 12 males, 20–60 years old) using a 9‐point hedonic scale (1 being disliked very much and 9 being liked very much). The panel members were chosen from people without egg allergies (Yaver and Bilgiçli 2021). Three‐digit random numbers were used to code the freshly cooked samples, and samples were presented on porcelain plates.

Members of the sensory panel were selected using a random voluntary process and were notified that they had the freedom to quit the research at any stage. Before conducting sensory analysis, participants were formally briefed on the research objectives and protocols; subsequently, informed consent was obtained for their involvement in the evaluations and the utilization of their anonymized data in academic publications.

### 2.9 Statistical Analysis

All measurements were made at least three times and the results were presented as mean ± standard deviation. SPSS (IBM version 28, Chicago, IL, USA) software was employed to determine significant differences using two‐way analysis of variance (ANOVA) at a 0.05 level of significance.

## 3 Results and Discussion

### 3.1 Impacts of Mucilage Type and Level on the Color Profile of Wheat Noodles

The appearance of a food product has an important effect on consumer choice. The color L*, a*, b* and ΔE values of wheat noodles are shown in Table S1. The impacts of mucilage type and level on the color profile are given in Table 1.

**TABLE 1:** Impacts of flaxseed mucilage type and level on the color profile of wheat noodles.

Factor	*n*	L*	a*	b*	Δ*E*
*Mucilage type*					
CFM	10	73.70 ± 0.66^a^	0.66 ± 0.70^a^	13.25 ± 3.39^a^	3.88 ± 3.53^b^
USFM	10	74.46 ± 1.03^a^	0.68 ± 0.67^a^	13.39 ± 3.46^a^	4.20 ± 3.51^a^
*Mucilage level (%)*					
Control	4	73.06 ± 0.08^b^	0.06 ± 0.04^d^	16.26 ± 0.08^a^	—
25	4	73.49 ± 0.38^b^	0.16 ± 0.01^d^	16.08 ± 0.12^a^	0.49 ± 0.24^d^
50	4	74.15 ± 0.79^ab^	0.41 ± 0.06^c^	14.28 ± 0.23^b^	2.34 ± 0.21^c^
75	4	74.51 ± 0.69^ab^	1.01 ± 0.01^b^	11.95 ± 0.02^c^	4.67 ± 0.24^b^
100	4	75.19 ± 0.74^a^	1.71 ± 0.02^a^	8.05 ± 0.02^d^	8.66 ± 0.21^a^

*Note:* Different lowercase letters show statistically significant variations in the same column (*p* < 0.05). ΔE, total color difference.

Abbreviations: CFM, conventionally extracted flaxseed mucilage; *n*, number of samples analyzed; USFM, ultrasonically extracted flaxseed mucilage.

The mean L*, a* and b* values of wheat noodles were 74.08, 0.67 and 13.32, respectively (Table S1). As illustrated in Table 1, mucilage type did not have a significant (*p* > 0.05) influence on the L*, a* and b* values of wheat noodles. Compared with CFM, the use of USFM resulted in higher ΔE values in noodles. This can be attributed to the lighter color of USFM due to its higher purity than CFM as a result of low extraction time in sonication (Yaver et al. 2026). Farahnaky et al. (2013) also reported that *Salvia macrosiphon* seed mucilage extracted by ultrasound had a lighter color than mucilage extracted by the conventional method, probably due to leaching impurities to mucilage solution in the conventional method.

Increasing flaxseed mucilage level from 0% to 100% led to an increase in the L* and a* values of noodles (Table 1). In contrast, the b* value of noodles exhibited a decreasing trend. These results may be attributed to the decreasing carotenoid content of noodles due to replacing eggs with flaxseed mucilage (Li et al. 2024). Shi et al. (2025) also reported that plant‐based gels using as egg replacers showed higher a* and lower b* values than whole eggs. The ΔE value increased with higher mucilage levels, showing a considerable color difference between the control and mucilage‐containing noodles (Table 1). As reported by Wang et al. (2025), an increase in a* value of noodles could positively affect consumer preference, especially those who want vibrant foods.

### 3.2 Impacts of Mucilage Type and Level on the Cooking and Textural Quality of Wheat Noodles

Water uptake, volume increase and cooking loss values, representing the cooking quality of noodles, are presented in Table S2. The mean water uptake, volume increase and cooking loss values of wheat noodles made with CFM and USFM were found to be 194.3%, 227.8%, and 4.86%, respectively.

Compared to noodles prepared with CFM, USFM noodles had a higher water uptake value (Table 2). According to Awuchi et al. (2019), the ultrasonication process may have altered the structure of polysaccharides in flaxseed mucilage, resulting in higher solubility and thus increasing its water absorption capacity. Therefore, greater water uptake values in USFM noodles (Table 2) could be related to the greater water absorption capacity of USFM than that of CFM (Yaver et al. 2026). Regarding the mucilage level factor, the greatest water uptake value was obtained in noodles produced from 50% of flaxseed mucilage (Table 2), probably due to an increase in soluble fiber content as a result of mucilage addition (Singh et al. 2025). As the level of soluble fibers increased, more water was absorbed and the water uptake value increased (Xiong et al. 2024). But the higher levels of mucilage reduced water uptake values (Table 2). The decrease may be due to the competition for water between soluble fibers in mucilage and starch. This competition inhibits water absorption and the expansion of starch molecules, reducing the water uptake of noodles (Jin et al. 2025). These findings were consistent with the study of Gupta et al. (2021), who reported that the resilience of the network generated by starch, protein, or fiber influenced the water absorption of noodles.

**TABLE 2:** Impacts of flaxseed mucilage type and level on the cooking and textural quality of wheat noodles.

Factor	*n*	Water uptake (%)	Volume increase (%)	Cooking loss (%)	Firmness (*N*)
*Mucilage type*					
CFM	10	193.1 ± 17.0^b^	217.5 ± 11.6^b^	4.93 ± 0.36^a^	4.85 ± 0.56^b^
USFM	10	195.5 ± 16.0^a^	238.1 ± 20.7^a^	4.80 ± 0.39^a^	5.09 ± 0.66^a^
*Mucilage level (%)*					
Control	4	181.1 ± 0.3^d^	215.6 ± 0.0^d^	5.32 ± 0.02^a^	4.30 ± 0.04^d^
25	4	206.7 ± 2.2^b^	240.6 ± 17.7^b^	5.14 ± 0.08^ab^	4.61 ± 0.19^c^
50	4	212.8 ± 0.9^a^	245.3 ± 24.3^a^	4.86 ± 0.17^bc^	4.83 ± 0.32^c^
75	4	196.8 ± 3.8^c^	228.1 ± 17.7^c^	4.58 ± 0.10^cd^	5.31 ± 0.13^b^
100	4	174.1 ± 3.5^e^	209.4 ± 13.3^e^	4.42 ± 0.09^d^	5.82 ± 0.25^a^

*Note:* Different lowercase letters show statistically significant variations in the same column (*p* < 0.05).

Abbreviations: CFM, conventionally extracted flaxseed mucilage; *n*, number of samples analyzed; USFM, ultrasonically extracted flaxseed mucilage.

As seen in Table 2, replacing eggs with USFM instead of CFM elicited higher volume increase values in wheat noodles. The ultrasound process can break the intracellular and intercellular covalent bonds in the cell wall, resulting in more OH groups, which improve the swelling power of mucilage (MacDougall and Ring 2003). The higher volume increase in noodle samples formulated with USFM may be associated with the greater swelling power of USFM than that of CFM (Yaver et al. 2026). An enhancement in the swelling power was also reported by Manchun et al. (2012) for tapioca starch after ultrasonic treatment. Compared with the control, the volume increase values of noodles first increased and then decreased as the level of flaxseed mucilage added increased (Table 2). The increase could be associated with the soluble fiber content of flaxseed mucilage (Kaushik et al. 2017). Similarly, Hymavathi et al. (2019) found an enhancement in volume increase values of wheat noodles when adding Karaya gum. However, the excessive addition of flaxseed mucilage reduced the protein content of noodles (Table 3), which might have weakened the protein crosslinking and resulted in lower swelling power (Jin et al. 2025).

**TABLE 3:** Impacts of flaxseed mucilage type and level on the chemical composition of wheat noodles.

Factor	*n*	Moisture (%)	Total ash (%)	Crude fat (%)	Crude protein (%)	Carbohydrate (%)	Energy (kcal/100 g)
*Mucilage type*							
CFM	10	7.36 ± 0.57^a^	1.16 ± 0.07^a^	2.01 ± 0.99^a^	14.60 ± 0.79^a^	74.87 ± 2.40^a^	376.0 ± 2.5^a^
USFM	10	7.41 ± 0.61^a^	1.12 ± 0.08^a^	1.75 ± 1.03^b^	14.76 ± 0.67^a^	74.96 ± 2.37^a^	374.6 ± 2.7^b^
*Mucilage level (%)*							
Control	4	7.98 ± 0.01^a^	1.24 ± 0.00^a^	3.16 ± 0.00^a^	15.65 ± 0.02^a^	71.98 ± 0.01^e^	378.9 ± 0.0^a^
25	4	7.88 ± 0.08^a^	1.19 ± 0.05^ab^	2.38 ± 0.39^b^	15.09 ± 0.10^b^	73.47 ± 0.25^d^	375.6 ± 2.1^b^
50	4	7.53 ± 0.08^b^	1.14 ± 0.04^bc^	1.96 ± 0.19^c^	14.66 ± 0.14^c^	74.72 ± 0.00^c^	375.1 ± 1.1^b^
75	4	6.94 ± 0.06^c^	1.09 ± 0.03^cd^	1.42 ± 0.16^d^	14.22 ± 0.17^d^	76.34 ± 0.03^b^	374.9 ± 0.9^b^
100	4	6.62 ± 0.06^d^	1.04 ± 0.03^d^	0.49 ± 0.18^e^	13.79 ± 0.19^e^	78.07 ± 0.08^a^	371.8 ± 0.6^c^

*Note:* Different lowercase letters show statistically significant variations in the same column (*p* < 0.05).

Abbreviations: CFM, conventionally extracted flaxseed mucilage; *n*, number of samples analyzed; USFM, ultrasonically extracted flaxseed mucilage.

As depicted in Table 2, there were no notable differences in cooking loss values among noodles made from CFM and USFM. This finding is consistent with previous results indicating that the use of lupin flour debittered by ultrasound‐assisted extraction elicited similar cooking loss values in pasta to conventionally debittered ones (Yaver and Bilgiçli 2021). Niu et al. (2014) also reported that ultrasound treatment did not considerably affect the cooking loss values of whole wheat noodles. On the other hand, replacing eggs with flaxseed mucilage (> 25%) considerably reduced the cooking loss values of wheat noodles (Table 2). When replacing eggs with flaxseed mucilage, increasing soluble fiber content could hinder full hydration of starch and decrease its solubility (Xiong et al. 2024; Bhuiyan et al. 2025), thus reducing cooking loss of noodles. Additionally, Singh et al. (2002) suggested that generating the interactions among amylose and mucilage could reduce the leaching of starch to boiling water. Kasunmala et al. (2020) also reported that increasing levels of xanthan gum from 0.1% to 5% in noodle formulations reduced cooking loss values from 8.97% to 7.40%.

Analysis of texture characteristics is essential in determining noodle quality because it affects consumer acceptance (Yang et al. 2021). Firmness of wheat noodles made with CFM and USFM ranged from 4.27 to 6.00 N (Table S2). The firmest texture was obtained in noodles produced with USFM (Table 2). This may be attributed to superior functional properties of USFM compared to CFM (Yaver et al. 2026), improving the viscous behavior of noodles (Kasunmala et al. 2020). Besides that, Niu et al. (2014) stated that there are no significant differences between the textural properties of control and ultrasound‐treated noodle samples. The differences in frequency, amplitude, and duration of sonication may be responsible for the differences between the present study and the literature. Regarding mucilage level, replacement of eggs with flaxseed mucilage drastically enhanced the firmness of noodles compared to the control (Table 2). A similar observation was reported by Fu et al. (2025) in their research on hydrophilic colloids and barley noodle quality. They attributed the enhanced cooking quality to the adhesive features of hydrophilic colloids, which encourage interlinking of molecules in the noodles, resulting in a firmer composition that endures cooking without falling apart (Sandhu et al. 2015). The data of the present study are encouraging and they validate the potential of flaxseed mucilage as a valuable egg replacer for noodles.

### 3.3 Impacts of Mucilage Type and Level on the Chemical Quality of Wheat Noodles

Moisture content of wheat noodles ranged from 6.58% to 7.98% (Table S3). The use of CFM or USFM as an egg replacer in wheat noodles did not elicit a considerable difference in the moisture level (Table 3). However, replacing 50%, 75%, and 100% of eggs with flaxseed mucilage resulted in lower moisture content in noodles compared to the control. This decrease could be due to the lower moisture content of flaxseed mucilage than eggs (Sharaf 2011; Yaver 2026).

The mean total ash contents of wheat noodles produced from CFM and USFM were 1.16% and 1.12%, respectively (Table 3). When the replacement of 25% of eggs with mucilage in noodles demonstrated similar ash content to the control, higher replacement levels reduced the ash content. The findings might be attributed to the utilization of mucilage suspension as an egg replacer in this study, resulting in lower ash content.

As shown in Table S3, fat contents of wheat noodles have changed in the range of 0.36% to 3.16%. The lower fat content found in USFM noodles compared to CFM noodles (Table 3) could be related to the presence of less fat in USFM (Yaver 2026). On the other hand, the fat content decreased when flaxseed mucilage was used as a substitute for eggs (Table 3). Higher fat content of eggs (Sharaf 2011) than flaxseed mucilage (Yaver 2026) may be responsible for these findings. Similar results were observed by Odep et al. (2024), who prepared mayonnaise with a lower fat content than the control by adding chia mucilage gel as an egg replacer.

Mucilage type did not have a remarkable influence on the protein content of wheat noodles (Table 3). However, increasing the level of mucilage in noodles gradually decreased protein content, which was probably due to the lower protein content of flaxseed mucilage (Yaver 2026) than that of eggs (Sharaf 2011). Besides that, the lowest protein content was found as 13.79% in noodles made with 100% mucilage (Table 3). Karpinska‐Tymoszczyk et al. (2021) found that when eggs were replaced with chia mucilage in the meatballs, the fat and protein contents of meatballs notably reduced, which was consistent with the findings of this study.

There was no considerable change between the two mucilage types in terms of carbohydrate content (Table 3). At a flaxseed mucilage addition amount of 100%, the carbohydrate content of wheat noodles reached its maximum, indicating a substantial enhancement in carbohydrate/soluble fiber content because of flaxseed mucilage incorporation (Singh et al. 2025). Soluble fibers are highly desirable in food products for human health as well as functional quality. It is known that seed mucilages represent relevant sources of soluble dietary fibers (Singh et al. 2025). The consumption of fiber‐rich foods could reduce blood pressure and cholesterol levels and the risks of obesity, diabetes, gastrointestinal and cardiovascular diseases (Ruhee and Suzuki 2018; Soliman 2019). Therefore, the high carbohydrate content of noodles made from flaxseed mucilages could offer many health benefits as well as unique rheological features (Capitani et al. 2015; Capriles et al. 2016).

Regarding mucilage type, the lowest energy value was obtained in noodles prepared from USFM (Table 3). The lower fat content of USFM than that of CFM (Yaver 2026) may be responsible for the lower energy values of USFM noodles. Compared to the control, replacement of eggs with flaxseed mucilage considerably reduced the energy values of noodles (Table 3). The lowest energy value was observed in noodles made from 100% flaxseed mucilage. Lower protein and fat contents of noodles containing mucilage than those of the control may have reduced the energy value. Abdelsatter et al. (2025) conducted a similar study in which proximate composition was measured in a cupcake. The results demonstrated that a decrease in fat content and energy value in the vegan cupcake is due to the replacement of eggs with chia mucilage.

### 3.4 Impacts of Mucilage Type and Level on the Antioxidant Properties of Wheat Noodles

Total phenolic content and antioxidant activity of wheat noodles are presented in Table S4, and the impacts of mucilage type and level on the antioxidant properties are illustrated in Table 4.

**TABLE 4:** Impacts of flaxseed mucilage type and level on the antioxidant properties of wheat noodles.

Factor	*n*	Total phenolic content (g GAE/kg)	Antioxidant activity (μmol TE/g)
*Mucilage type*			
CFM	10	0.42 ± 0.02^a^	1.90 ± 0.01^a^
USFM	10	0.42 ± 0.02^a^	1.93 ± 0.03^a^
*Mucilage level (%)*			
Control	4	0.43 ± 0.01^a^	1.91 ± 0.00^a^
25	4	0.42 ± 0.01^ab^	1.92 ± 0.00^a^
50	4	0.42 ± 0.00^ab^	1.92 ± 0.02^a^
75	4	0.41 ± 0.01^b^	1.91 ± 0.03^a^
100	4	0.39 ± 0.03^c^	1.94 ± 0.06^a^

*Note:* Different lowercase letters show statistically significant variations in the same column (*p* < 0.05).

Abbreviations: CFM, conventionally extracted flaxseed mucilage; GAE, gallic acid equivalent; *n*, number of samples analyzed; TE, trolox equivalent; USFM, ultrasonically extracted flaxseed mucilage.

Total phenolic content of wheat noodles ranged from 0.39 to 0.43 g GAE/kg (Table S4). Wheat noodles made from CFM had a similar phenolic content to noodles from USFM (Table 4). Yaver and Bilgiçli (2021) also stated that the antioxidant properties of pasta made with debittered lupin flour by ultrasound were statistically similar (*p* > 0.05) to those of pasta made with conventionally debittered lupin flour. Moreover, replacing 25% and 50% of eggs with mucilage in noodle samples elicited similar (*p* > 0.05) phenolic content to the control (Table 4). But the higher levels of flaxseed mucilage slightly reduced the total phenolic content of noodles.

Regarding antioxidant activity, USFM demonstrated no statistically significant (*p* > 0.05) change with that of CFM (Table 4). Furthermore, the replacement of eggs with flaxseed mucilage did not elicit any considerable difference in the antioxidant activity of wheat noodles. Similar observations were also found for total phenolic content and DPPH radical scavenging activity by Freire et al. (2024) when adding xanthan gum to pasta. The findings showed that flaxseed mucilage used as an egg replacer compensated for the antioxidant impacts in eggless wheat noodles.

### 3.5 Impacts of Mucilage Type and Level on the Sensory Quality of Wheat Noodles

Sensory assessment is crucial for comprehending consumer likes and acts as an essential instrument in product innovation. Sensory evaluation results of wheat noodles, including parameters taste, odor, color, appearance, mouthfeel, and overall acceptability are presented in Table S5 and the impacts of mucilage type and level on the mentioned parameters are shown in Table 5.

**TABLE 5:** Impacts of flaxseed mucilage type and level on the sensory quality of wheat noodles.

Factor	*n*	Taste	Odor	Color	Appearance	Mouthfeel	Overall acceptability
*Mucilage type*							
CFM	10	7.84 ± 0.70^b^	7.73 ± 0.74^a^	7.44 ± 0.75^a^	7.50 ± 0.49^a^	7.46 ± 0.32^a^	7.39 ± 0.63^b^
USFM	10	8.13 ± 0.42^a^	7.96 ± 0.60^a^	7.37 ± 0.78^a^	7.60 ± 0.23^a^	7.58 ± 0.40^a^	7.68 ± 0.44^a^
*Mucilage level (%)*							
Control	4	8.48 ± 0.04^a^	8.48 ± 0.04^a^	8.28 ± 0.04^a^	8.00 ± 0.00^a^	7.00 ± 0.00^c^	8.03 ± 0.04^a^
25	4	8.45 ± 0.07^a^	8.35 ± 0.07^a^	8.00 ± 0.00^a^	7.65 ± 0.21^ab^	7.35 ± 0.07^bc^	7.95 ± 0.21^a^
50	4	8.15 ± 0.21^a^	7.95 ± 0.21^b^	7.45 ± 0.07^b^	7.55 ± 0.21^bc^	7.55 ± 0.07^ab^	7.70 ± 0.14^a^
75	4	7.70 ± 0.28^b^	7.65 ± 0.21^b^	6.85 ± 0.07^c^	7.35 ± 0.21^bc^	7.85 ± 0.07^a^	7.25 ± 0.35^b^
100	4	7.15 ± 0.49^c^	6.80 ± 0.28^c^	6.45 ± 0.07^d^	7.20 ± 0.57^c^	7.85 ± 0.21^a^	6.75 ± 0.35^c^

*Note:* Different lowercase letters show statistically significant variations in the same column (*p* < 0.05).

Abbreviations: CFM, conventionally extracted flaxseed mucilage; *n*, number of samples analyzed; USFM, ultrasonically extracted flaxseed mucilage.

As shown in Table 5, the mucilage type factor considerably influenced taste and overall acceptability scores of wheat noodles. Noodles made from USFM had greater taste and overall acceptability scores. The superior cooking and textural quality of USFM noodles compared to CFM‐containing counterparts (Table 2) may be responsible for these findings (Table 5). However, there are no notable differences between the odor, color, appearance, and mouthfeel scores of noodles made from CFM and USFM.

Regarding the mucilage level factor, taste scores of noodles made from 25% and 50% of mucilage were similar (*p* > 0.05) to the control (Table 5). Furthermore, the replacement of 25% of eggs with flaxseed mucilage did not result in significant (*p* > 0.05) alterations in other sensory scores. Compared to the control, noodles produced from 50% and 75% of mucilage slightly decreased odor, color, and appearance scores but enhanced mouthfeel. In terms of overall acceptability, noodles prepared with 25%, 50%, and 75% flaxseed mucilage had a score above 7. In total, 100% of mucilage noodles received the lowest taste (7.15), odor (6.80), color (6.45), and overall acceptability (6.75) scores. The deterioration in the cooking quality of noodles (Table 2) could have contributed to the reduced sensory scores (except for mouthfeel) at elevated mucilage levels (Table 5). Similarly, Vu et al. (2025) found that complete replacement of eggs with chia gel considerably reduced sensory scores of brownies.

## 4 Conclusions

This study examined the impacts of the replacement of eggs with flaxseed mucilages obtained by conventional and ultrasound‐assisted methods at different levels on wheat noodle quality. Replacing eggs with USFM improved the cooking and textural quality of noodles in comparison with CFM. Moreover, USFM noodles had a lower energy value and higher overall acceptability than CFM noodles. On the other hand, results showed that partial replacement (25%, 50%, and 75%) of eggs with flaxseed mucilage considerably improved cooking quality and texture of wheat noodles. Besides that, noodles made from 100% mucilage had a lighter color, firmer texture and lower energy value than 100% egg noodles. However, replacing eggs with flaxseed mucilage reduced the ash, protein and phenolic contents of noodles. In addition, there are no substantial differences between the overall acceptability scores of the control and noodles containing 25% and 50% of flaxseed mucilage. This study provides support for expanding the use of flaxseed mucilage (especially obtained by ultrasound‐assisted extraction) as a potential egg replacer (up to 75%) in wheat noodles with satisfactory technological and sensory quality.

## Author Contributions

**Elif Yaver:** conceptualization, investigation, funding acquisition, writing – original draft, methodology, writing – review and editing, formal analysis, project administration, supervision, resources.

## Funding

This work was supported by the Scientific Research Project Fund of Konya Technical University (grant number 232022043).

## Ethics Statement

Members of the sensory panel were selected using a random voluntary process and were notified that they had the freedom to quit the research at any stage. Before conducting sensory analysis, participants were formally briefed on the research objectives and protocols; subsequently, informed consent was obtained for their involvement in the evaluations and the utilization of their anonymized data in academic publications.

## Conflicts of Interest

The author declares no conflicts of interest.

## Supporting information


**Table S1:** Color values of wheat noodles.
**Table S2:** Cooking and textural properties of wheat noodles.
**Table S3:** Chemical composition of wheat noodles.
**Table S4:** Antioxidant properties of wheat noodles.
**Table S5:** Sensory quality of wheat noodles.

## Data Availability

The data are available from the author upon reasonable request.
