# Supplementary material for: Comparing Effects of Conventionally and Ultrasonically Extracted Flaxseed Mucilages as Sustainable Egg Replacers on the Technological Properties and Nutritional Quality of Dried Wheat Noodles
Source: Food Sci Nutr. 2026 Apr 2;14(4):e71727. doi: 10.1002/fsn3.71727 (PMC13052323; doi:10.1002/fsn3.71727)
Supplement: Supplementary file 1 — Table S1: Color values of wheat noodles. Table S2: Cooking and textural properties of wheat noodles. Table S3: Chemical composition of wheat noodles. Table S4: Antioxidant properties of wheat noodles. Table S5: Sensory quality of wheat noodles. [file FSN3-14-e71727-s001.doc]

**Comparing effects of conventionally and ultrasonically extracted flaxseed mucilages as sustainable egg replacers on the technological properties and nutritional quality of dried wheat noodles**

Elif Yaver*

Department of Food Processing, Vocational School of Technical Sciences, Konya Technical University, 42250, Selçuklu, Konya, Türkiye.

[elifyaver@hotmail.com](mailto:elifyaver@hotmail.com) / https://orcid.org/0000-0002-2651-9922

***Corresponding author**

*Elif Yaver*

Address: Department of Food Processing, Vocational School of Technical Sciences, Konya Technical University, 42250, Selçuklu, Konya, Türkiye.

E-mail: elifyaver@hotmail.com

Phone: +90-332-223 23 78; Fax: +90-332-241 01 85

**Supporting Information**

**Table S1.** Color values of wheat noodles.

| Mucilage type | Mucilage level (%) | *L** | *a** | *b** | ΔE |
| --- | --- | --- | --- | --- | --- |
| CFM | Control | 73.01±0.89 | 0.04±0.02 | 16.20±0.75 | - |
|  | 25 | 73.22±0.92 | 0.15±0.04 | 15.99±0.92 | 0.32±0.05 |
|  | 50 | 73.59±0.83 | 0.36±0.08 | 14.11±0.82 | 2.19±0.12 |
|  | 75 | 74.02±0.92 | 1.01±0.13 | 11.93±0.72 | 4.49±0.15 |
|  | 100 | 74.67±0.88 | 1.72±0.17 | 8.03±0.88 | 8.50±0.21 |
| USFM | Control | 73.12±0.91 | 0.09±0.03 | 16.32±0.79 | - |
|  | 25 | 73.76±0.93 | 0.17±0.02 | 16.16±0.96 | 0.66±0.09 |
|  | 50 | 74.71±0.82 | 0.45±0.06 | 14.44±0.83 | 2.49±0.07 |
|  | 75 | 75.00±0.98 | 1.00±0.16 | 11.96±0.72 | 4.83±0.14 |
|  | 100 | 75.71±0.86 | 1.69±0.13 | 8.06±0.75 | 8.80±0.17 |
| Minimum-maximum | | 73.01-75.71 | 0.04-1.72 | 8.03-16.32 | 0.32-8.80 |
| Mean value±std | | 74.08±1.10 | 0.67±0.63 | 13.32±3.20 | 4.04±3.28 |

The values represent the average of three determinations from duplicate samples. CFM, Conventionally extracted flaxseed mucilage; USFM, Ultrasonically extracted flaxseed mucilage; ΔE, Total color difference.

**Table S2.** Cooking and textural properties of wheat noodles.

| Mucilage type | Mucilage level (%) | Water uptake  (%) | Volume increase  (%) | Cooking loss  (%) | Firmness  (N) |
| --- | --- | --- | --- | --- | --- |
| CFM | Control | 181.3±1.8 | 215.6±3.0 | 5.33±0.17 | 4.33±0.16 |
|  | 25 | 205.2±1.4 | 228.1±2.7 | 5.19±0.16 | 4.47±0.18 |
|  | 50 | 213.5±2.5 | 228.1±1.5 | 4.98±0.14 | 4.60±0.15 |
|  | 75 | 194.1±1.6 | 215.6±1.9 | 4.65±0.21 | 5.21±0.19 |
|  | 100 | 171.7±1.2 | 200.0±2.8 | 4.48±0.18 | 5.64±0.20 |
| USFM | Control | 180.8±1.9 | 215.6±2.3 | 5.30±0.20 | 4.27±0.16 |
|  | 25 | 208.2±1.7 | 253.1±1.8 | 5.08±0.16 | 4.73±0.16 |
|  | 50 | 212.2±2.4 | 262.5±2.1 | 4.74±0.18 | 5.05±0.22 |
|  | 75 | 199.5±1.9 | 240.6±2.0 | 4.51±0.17 | 5.40±0.15 |
|  | 100 | 176.6±1.2 | 218.8±2.0 | 4.35±0.21 | 6.00±0.21 |
| Minimum-maximum | | 171.7-213.5 | 200.0-262.5 | 4.35-5.33 | 4.27-6.00 |
| Mean value±std |  | 194.3±15.3 | 227.8±18.7 | 4.86±0.37 | 4.97±0.59 |

The values represent the average of three determinations from duplicate samples. CFM, Conventionally extracted flaxseed mucilage; USFM, Ultrasonically extracted flaxseed mucilage.

**Table S3. Chemical composition of wheat noodles.**

| Mucilage type | Mucilage level (%) | Moisture  (%) | Total ash  (%) | Crude fat  (%) | Crude protein  (%) | Carbohydrate  (%) | Energy  (kcal/100 g) |
| --- | --- | --- | --- | --- | --- | --- | --- |
| CFM | Control | 7.97±0.07 | 1.24±0.05 | 3.16±0.08 | 15.67±0.14 | 71.97±0.46 | 379.0±2.0 |
|  | 25 | 7.82±0.06 | 1.22±0.07 | 2.66±0.08 | 15.01±0.18 | 73.29±0.30 | 377.1±1.2 |
|  | 50 | 7.47±0.09 | 1.16±0.06 | 2.09±0.06 | 14.55±0.21 | 74.72±0.34 | 375.9±0.9 |
|  | 75 | 6.90±0.11 | 1.11±0.03 | 1.53±0.07 | 14.10±0.16 | 76.36±0.37 | 375.6±1.2 |
|  | 100 | 6.66±0.10 | 1.06±0.04 | 0.62±0.04 | 13.65±0.13 | 78.01±0.44 | 372.2±1.3 |
| USFM | Control | 7.98±0.11 | 1.24±0.06 | 3.16±0.06 | 15.64±0.14 | 71.98±0.33 | 378.9±1.6 |
|  | 25 | 7.94±0.07 | 1.15±0.05 | 2.10±0.04 | 15.16±0.20 | 73.65±0.39 | 374.1±1.1 |
|  | 50 | 7.59±0.09 | 1.11±0.04 | 1.82±0.07 | 14.76±0.17 | 74.73±0.35 | 374.3±1.3 |
|  | 75 | 6.98±0.07 | 1.07±0.07 | 1.30±0.04 | 14.34±0.13 | 76.31±0.45 | 374.3±1.1 |
|  | 100 | 6.58±0.06 | 1.02±0.04 | 0.36±0.03 | 13.92±0.16 | 78.12±0.34 | 371.4±0.9 |
| Minimum-maximum | | 6.58-7.98 | 1.02-1.24 | 0.36-3.16 | 13.65-15.67 | 71.97-78.12 | 371.4-379.0 |
| Mean value±std | | 7.39±0.55 | 1.14±0.08 | 1.88±0.94 | 14.68±0.69 | 74.91±2.21 | 375.3±2.6 |

The values represent the average of three determinations from duplicate samples. CFM, Conventionally extracted flaxseed mucilage; USFM, Ultrasonically extracted flaxseed mucilage.

**Table S4. Antioxidant properties of wheat noodles.**

| Mucilage type | Mucilage level (%) | Total phenolic content  (g GAE/kg) | Antioxidant activity  (µmol TE/g) |
| --- | --- | --- | --- |
| CFM | Control | 0.43±0.01 | 1.91±0.07 |
|  | 25 | 0.43±0.01 | 1.92±0.04 |
|  | 50 | 0.42±0.01 | 1.90±0.05 |
|  | 75 | 0.41±0.01 | 1.89±0.06 |
|  | 100 | 0.39±0.01 | 1.89±0.03 |
| USFM | Control | 0.43±0.01 | 1.91±0.03 |
|  | 25 | 0.42±0.01 | 1.92±0.06 |
|  | 50 | 0.42±0.01 | 1.93±0.07 |
|  | 75 | 0.41±0.01 | 1.93±0.04 |
|  | 100 | 0.39±0.01 | 1.98±0.03 |
| Minimum-maximum |  | 0.39-0.43 | 1.89-1.98 |
| Mean value±std |  | 0.42±0.02 | 1.92±0.04 |

The values represent the average of three determinations from duplicate samples. CFM, Conventionally extracted flaxseed mucilage; USFM, Ultrasonically extracted flaxseed mucilage; GAE, Gallic acid equivalent; TE, Trolox equivalent.

**Table S5.** Sensory quality of wheat noodles.

| Mucilage type | Mucilage level (%) | Taste | Odor | Color | Appearance | Mouthfeel | Overall acceptability |
| --- | --- | --- | --- | --- | --- | --- | --- |
| CFM | Control | 8.50±0.21 | 8.45±0.28 | 8.30±0.25 | 8.00±0.23 | 7.00±0.28 | 8.05±0.24 |
|  | 25 | 8.40±0.25 | 8.30±0.21 | 8.00±0.27 | 7.80±0.28 | 7.30±0.25 | 7.80±0.27 |
|  | 50 | 8.00±0.28 | 7.80±0.21 | 7.50±0.23 | 7.70±0.21 | 7.50±0.27 | 7.60±0.25 |
|  | 75 | 7.50±0.24 | 7.50±0.24 | 6.90±0.23 | 7.20±0.25 | 7.80±0.21 | 7.00±0.21 |
|  | 100 | 6.80±0.23 | 6.60±0.27 | 6.50±0.24 | 6.80±0.24 | 7.70±0.23 | 6.50±0.28 |
| USFM | Control | 8.45±0.21 | 8.50±0.20 | 8.25±0.21 | 8.00±0.27 | 7.00±0.25 | 8.00±0.27 |
|  | 25 | 8.50±0.27 | 8.40±0.21 | 8.00±0.28 | 7.50±0.25 | 7.40±0.27 | 8.10±0.20 |
|  | 50 | 8.30±0.24 | 8.10±0.27 | 7.40±0.20 | 7.40±0.21 | 7.60±0.25 | 7.80±0.24 |
|  | 75 | 7.90±0.21 | 7.80±0.20 | 6.80±0.24 | 7.50±0.25 | 7.90±0.21 | 7.50±0.21 |
|  | 100 | 7.50±0.28 | 7.00±0.25 | 6.40±0.27 | 7.60±0.28 | 8.00±0.24 | 7.00±0.24 |
| Minimum-maximum | | 6.80-8.50 | 6.60-8.50 | 6.40-8.30 | 6.80-8.00 | 7.00-8.00 | 6.50-8.10 |
| Mean value±std | | 7.99±0.58 | 7.85±0.65 | 7.40±0.72 | 7.55±0.40 | 7.52±0.38 | 7.54±0.55 |

The values represent the average of three determinations from duplicate samples. CFM, Conventionally extracted flaxseed mucilage; USFM, Ultrasonically extracted flaxseed mucilage.
